# Caffeic Acid Targets AMPK Signaling and Regulates Tricarboxylic Acid Cycle Anaplerosis while Metformin Downregulates HIF-1α-Induced Glycolytic Enzymes in Human Cervical Squamous Cell Carcinoma Lines

**DOI:** 10.3390/nu10070841

**Published:** 2018-06-28

**Authors:** Malgorzata Tyszka-Czochara, Karolina Bukowska-Strakova, Kinga A. Kocemba-Pilarczyk, Marcin Majka

**Affiliations:** 1 Department of Food Chemistry and Nutrition, Faculty of Pharmacy, Jagiellonian University Medical College, Medyczna 9, 30-688 Krakow, Poland; 2 Department of Clinical Immunology, Institute of Pediatrics, Jagiellonian University Medical College, Wielicka 265, 30-663 Krakow, Poland; k.bukowska-strakova@uj.edu.pl; 3 Faculty of Medicine, Jagiellonian University-Medical College, Kopernika 7, 31-034 Krakow, Poland; kinga.kocemba@uj.edu.pl; 4 Department of Department of Transplantation, Faculty of Medicine, Jagiellonian University Medical College, Wielicka 258, 30-688 Krakow, Poland

**Keywords:** cancer metabolism, metabolic reprogramming, cervical cancer, Warburg effect, 5′-adenosine monophosphate-activated protein kinase (AMPK), Caffeic Acid, Metformin, polyphenols, mitochondria

## Abstract

The small molecules, natural antioxidant Caffeic Acid (trans-3,4-Dihydroxycinnamic acid CA) and anti-diabetic drug Metformin (Met), activate 5′-adenosine monophosphate-activated protein kinase (AMPK) and interfere with metabolic reprogramming in human cervical squamous carcinoma cells. Here, to gain more insight into the ability of CA, Met and the combination of both compounds to impair aerobic glycolysis (the “Warburg effect”) and disrupt bioenergetics of cancer cells, we employed the cervical tumor cell lines C-4I and HTB-35/SiHa. In epithelial C-4I cells derived from solid tumors, CA alleviated glutamine anaplerosis by downregulation of Glutaminase (GLS) and Malic Enzyme 1 (ME1), which resulted in the reduction of NADPH levels. CA treatment of the cells altered tricarboxylic acid (TCA) cycle supplementation with pyruvate via Pyruvate Dehydrogenase Complex (PDH), increased ROS formation and enhanced cell death. Additionally, CA and CA/Met evoked intracellular energetic stress, which was followed by activation of AMPK and the impairment of unsaturated FA de novo synthesis. In invasive HTB-35 cells, Met inhibited Hypoxia-inducible Factor 1 (HIF-1α) and suppressed the expression of the proteins involved in the “Warburg effect”, such as glucose transporters (*GLUT1*, *GLUT3*) and regulatory enzymes of glycolytic pathway Hexokinase 2 (*HK2*), 6-Phosphofructo-2-Kinase/Fructose-2,6-Biphosphatase 4 (*PFKFB4*), Pyruvate Kinase (*PKM*) and Lactate Dehydrogenase A (*LDH*). Met suppressed the expression of *c-Myc*, *BAX* and cyclin-D1 (*CCND1*) and evoked apoptosis in HTB-35 cells. In conclusion, both small molecules CA and Met are capable of disrupting energy homeostasis, regulating oxidative metabolism/glycolysis in cervical tumor cells in regard to specific metabolic phenotype of the cells. CA and Met may provide a promising approach in the prevention of cervical cancer progression.

## 1. Introduction

Recently, the reprogramming of cancer cell metabolism has been acknowledged as a promising target for pharmacological interventions [1]. The inhibition of selected key points regulating biosynthetic pathways with regard to specific metabolic phenotype of tumor cell may help to establish specific anticancer approaches [2,3]. The activation of main sensor of energy status 5′-adenosine monophosphate-activated protein kinase (AMPK) by acting on cell cycle checkpoints and suppressing biosynthesis influences cell survival [4]. Depending on cellular context, the activation or silencing of AMPK regulates metabolic pathways which in turn affects proliferation of neoplastic cells [5]. The role of AMPK in cervical cancer development and progression has been only partially recognized. However, emerging data have suggested, that relevant manipulation of AMPK signaling in tumors may selectively induce cell death.

The anticancer potential of small molecules that can interfere with cellular bioenergetics by depletion of ATP and regulate biosynthetic processes via AMPK signaling have been recently intensively studied. Several natural compounds, such as Caffeic Acid (trans-3,4-Dihydroxycinnamic acid, CA), were reported to act via AMPK signaling [6]. CA is a major representative of hydroxycinnamic acids and appears in plants mainly as Chlorogenic Acid (5-caffeoylquinic acid, an ester of Caffeic Acid with Quinic Acid) [7]. Anti-tumor effects of CA have been recently demonstrated in breast cancer cells [8]. In colorectal carcinoma cells CA derivatives, involving the activation of AMPK and its downstream targets, inhibited the growth of cancer cells by modulation of phosphatidylinositide 3-kinases (PI3-K)/Akt and mammalian target of rapamycin (m-TOR) signaling pathways [9,10]. Biguanide Metformin (Met), has been reported to inhibit proliferation of numerous cancer cell lines [11,12]. Met was shown to decrease tumor growth and metastasis formation in animal models as well as in humans [13,14]. Mechanistic study revealed, that Met affects energy metabolism and exerts its effect by inhibition of Complex I of Electron Transport Chain (ETC) in mitochondria, which results in ATP depletion and remodeling of the network of biosynthetic processes within the cell [15]. Emerging data suggest that CA as well as Met can synergize with targeted anticancer therapies and enhance therapeutic response to drugs in specific tumor types [11,16,17,18].

The current strategies against cervical malignancy are being developed with regard not only to specific genetic background but also to metabolic specificity of cancer cells [19,20,21]. Numerous studies have shown that aerobic glycolysis generates ATP and provides substantial amounts of energy for aggressive tumors without excessive generation of Reactive Oxygen Species (ROS) [22]. Recently, we have found that HTB-35 cervical squamous carcinoma cells exhibit invasive phenotype and upregulated expression of mesenchymal genes [23], thus in the present study we aimed to explore whether CA and/or Met may influence the regulation of glycolytic pathway within HTB-35 cells. Since the activation of oncogene c-Myc and Hypoxia-inducible Factor 1 (HIF-1α) may trigger expression of glycolytic genes, we tested if CA and Met affect the expression of c-Myc and the function of HIF1-α in cells cultured in normoxic as well as hypoxic conditions. Furthermore, in order to find out how the drugs regulate mitochondrial metabolism of tumor cells with epithelial phenotype, we employed human cervical squamous carcinoma cell line C-4I, derived from in situ tumor, which expresses high genetic and metabolic agreement with cervical cancer biopsies [23,24]. Previously we demonstrated that CA and Met are capable of regulating mitochondrial TCA cycle supply in HTB-35 cell [18] and in HTB-34 cells derived from metastatic site [25]. Here, using C-4I cells we investigated, whether the regulation of pyruvate/glutamine-based supply of tricarboxylic acid (TCA) cycle may be relevant metabolic hot spot to affect cellular bioenergetics and biosynthetic pathways in epithelial tumor cells and we tested if such intervention results in the suppression of ATP/NADPH synthesis and further elimination of cervical cancer cells. As CA activated AMPK in HTB-35 cells, which undergo Epithelial-to-Mesenchymal Transition (EMT) [18] and in HTB-34 cells [25], which undergo EMT and subsequent Mesenchymal-to-Epithelial Transition (MET), we aimed to elucidate if CA and/or Met activate AMPK and regulate de novo synthesis/desaturation of fatty acids (FA) in C-4I tumor cells. We discussed the possibility of application of CA, Met and co-treatment as a therapeutic strategy for counteracting proliferation of cervical cancer with regard to bioavailability of tested compounds.

## 2. Materials and Methods

### 2.1. Cell Culture

Both cell lines C-4I (ATCC designation CRL-1594, human) and HTB-35 (ATCC designation HTB-35, SiHa, human) were derived from the American Type Cell Culture collection. C-4I line was maintained at 37 °C as a monolayer culture in Waymouth’s MB 752⁄1 medium (Life Technologies, New York, NY, USA). HTB-35 cells were grown in Dulbecco’s modified Eagle’s medium (Lonza, Walkersville, MD, USA) in atmosphere of 5% CO_2,_ unless otherwise noted. 10% *v*/*v* FBS (Eurex Sp z o.o., Gdansk, Poland) was used for media supplementation. 50 µg/mL of gentamicin was added to culture media (Sigma-Aldrich, Seelze, Germany). Cells up to the 25th passage were used. Trypsin-EDTA solution was used for subcultures. C-4I were seeded at a density of 2.5 × 10^5^ cells/mL and HTB-35 cells were seeded at a density of 1 × 10^5^ cells/mL into the 6-well plates (Sarstedt, Numbrecht, Germany) and incubated to archive the sufficient confluency for experiments. The cells were kept for 24 h in medium containing 0.5% *v*/*v* of bovine serum albumin (BSA, Sigma-Aldrich) and antibiotic. Then medium was changed for the new one serum-free Waymouth’s/0.5% BSA with adequate volumes of a stock solution of Met (10 mM, Sigma-Aldrich), CA (100 µM, Sigma-Aldrich) or Met (10 mM) and CA (100 µM) together. The cells were exposed to compounds for 24 h. The solvents of Met (PBS, Lonza) and CA (dimethyl sulfoxide, DMSO, 1% *v*/*v*, Sigma-Aldrich) were added to appropriate control wells. Each experiment was repeated three times. The cells as well as media were collected after each experiment. The automatic cell counter Countess (Gibco Laboratories, Grand Islands, NY, USA) was used for a precise measurement of cells number. The inverted light microscope Olympus CKX 41SF-5 (Olympus, Germany) was used for the inspection of the morphology of cell culture.

### 2.2. Flow Cytometry Analyses

For flow cytometry analyses, C-4I cells were plated in 6-well plates (Sarstedt) in triplicates at a density of 2.5 × 10^5^ cells per well. After exposure for 24 h to CA, Met and CA/Met, the cells were detached using Trypsin-EDTA solution, washed with buffered PBS (Lonza, Walkersville, MD, USA) and centrifuged at 350× *g* for 5 min. Then the cells were suspended in binding buffer at a room temperature. Fluorescent dyes, 488-AnnexinV (Biotium, Hayward, CA, USA; excitation maximum at 490 nm/emission maximum at 515 nm) and/or Ethidium homodimer (EthD-III, Biotium, CA, USA; excitation maximum at 528 nm/emission maximum at 617 nm) were added to cells’ suspension according to the manufacturer’s procedure. In order to correct discrimination between cells and debris, SYTO 41 Blue Fluorescent Nucleic Acid Stain was used (excitation maximum at 483 nm/emission maximum at 503 nm). The appropriate controls fluorescence minus one were prepared. The cells were incubated in dark for 15 min and acquired flow cytometer FACSCanto10C with BD FACSCanto System Software (BD Biosciences Immunocytometry Systems, San Jose, CA, USA). The cells were gated according to forward (FSC), side scatter (SSC) and fluorescence parameters (FITC channel was used for 488-AnnexinV and Texas Red channel was used for EthD-III). The details of analysis were described in [26]. The results were given as the percentage of apoptotic or necrotic cells of total counted cells. Simultaneously, the generation of mitochondrial superoxide was measured with MitoSox Red reagent (Invitrogen, CA, USA; excitation maximum at 510 nm/emission maximum at 580 nm) using FACSCanto10C cytometer (BD Biosciences). The cells were incubated for 10 min at 37 °C with 5 µM of reagent working solution prepared in DMSO.

### 2.3. Immunoblots

Cells for immunoblot analysis were incubated with appropriate concentrations of compounds in 6-well plates (Sarstedt) and homogenized in M-PER buffer (4 °C, Thermo Fisher Scientific Inc., Waltham, MA, USA). A mixture of water-soluble protease inhibitors (Merck, Darmstadt, Germany) was used to prevent proteolytic degradation of protein samples during cell lysis and extraction. Protein extracts were mixed with 4 Laemmli sample buffer and heated for 10 min., loaded onto an SDS gel, resolved via standard SDS-PAGE and, finally, transferred to PVDF membranes for Western blotting. The buffer used for membranes blocking with 1% BSA in Tris Buffered Saline with Tween 20 (TBST, pH 7.5). TBST contained 20 mM of Tris-hydrochloride, 0.05% Tween 20 and 150 mM NaCl (BioRad, Laboratories, Richmond, CA, USA), as earlier reported [18,25]. After being blocked, the membranes were probed for 12 h in buffer with addition of 1% BSA, 0.1% Tween 20 and the appropriate primary antibody. The immunodetection was performed using primary antibodies obtained from the following sources: anti-AMPK (Cell signaling, Danvers, MA, USA), anti-p-AMPK (Cell signaling), anti-p-PDH (Abcam, Cambridge, MA, USA), anti-PDH (Cell signaling), anti-CPT1 (Cell signaling), anti-GLUT1 (Santa Cruz Biotech., Santa Cruz, CA, USA), anti-p-ACC1 (Cell signaling), anti-ACC1 (Cell signalling) anti-ACLY, anti-FAS (Santa Cruz Biotech.), anti-SCD1(Santa Cruz Biotech.), anti-PDK-1 (Sigma-Aldrich, St Louis, MO, USA), anti-HK2 (Santa Cruz Biotch), anti-PKM2 (Sigma-Aldrich, MO, USA), anti-GLUT3 (Sigma-Aldrich, MO, USA) and anti-PFKFB4 (Abcam, MA, USA). β-actin (Cell signaling) was applied as the control of the loading process. The secondary antibodies conjugated to the horseradish peroxidase were from Santa Cruz Biotech. The protein expression was assayed using the Super Signal West Pico Chemiluminescent Substrate Kit, Pierce Chemical, Rockford, IL, USA). Gel Logic Imaging System 1500 (Kodak; Molecular imaging System Corestea Health Inc., Rochester, NY, USA) was used for the detection and analysis of the chemiluminescence signal. Bradford method was used for the measurement of the total protein amount, as described elsewhere. For HIF-1α analysis total protein was measured by of Lowry assay with modification of Peterson [23].

### 2.4. Pyruvate Dehydrogenase Kinase (PDK) Activity Assay

C-4I cells were exposed to Met, CA and Met/CA as described in Section 2.1. and then mitochondria were isolated from cells and processed as described previously [25]. For the assay, 20 mM Tris-hydrochloride buffer (pH 7.4; 2 mM EGTA, 5 mM K_2_HPO_4_, 120 mM KCl) containing 10 µM of uncoupler carbonyl cyanide-chlorophenylhydrazone (CCCP, Sigma-Aldrich) was used. The extraction buffer contained 0.5% *v*/*v* Triton-X100, 2 mM DTT, 50 mM K_2_HPO_4_, 10 mM EGTA, and 1% *v*/*v* BSA (Sigma-Aldrich). PDK activity was expressed as the rate of ATP-dependent inactivation of PDH complex and calculated as the apparent first-order rate constant.

### 2.5. Quantitation of NADP and NADPH Levels

Cells at a density of 2.5 × 10^5^ cells/mL were seeded in T-75 plates (Sarstedt), cultured and incubated with CA, Met and Met/CA for 24 h. Then the cells were harvested and homogenized according to manufacturer’s protocol. NADPH colorimetric quantitation kit was used to assess the intracellular concentrations of NADPH following deprotenization of samples by filtering through a 10 kDa cut-off filters. The absorbance was measured at 450 nm using microplate reader Infinite M200 Pro (Tecan, Salzburg, Austria).

### 2.6. Quantitation of ATP and ADP Levels

C-4I cells were placed in 96-well microtiter plate (Sarstedt) at a density of 2.5 × 10^4^ cells per well, cultured and then exposed to CA, Met and Met/CA co-treatment for 24 h. ADP/ATP ratio assay kit was employed to measure intracellular concentration of ATP and ADP in each sample. The procedure was conducted according to manufacturer’s protocol and luminescence (relative light units) was read using microplate reader (Tecan, Salzburg, Austria).

### 2.7. Hypoxia Conditions

The cells were seeded into plates (C4-I cells at a density of 2.5 × 10^5^ cells/mL and HTB-35 cells at a density of 1 × 10^5^ cells/mL) and cultivated in atmosphere of 5% CO_2_ at 37 °C until desired cell confluence was reached. Then the cells were exposed to CA, Met and CA/Met for 24 h under normoxic (oxygen concentration 21%) or hypoxic conditions (oxygen concentration 5%; a gas mixture contained 5% CO_2_ and a balance of nitrogen). The cells were kept in Panasonic MCO-170M-PE incubator (Gunma, Japan). Then the cells were harvested for RNA isolation [23].

### 2.8. Quantitative Polymerase Chain Reaction (qPCR)

Prior to RNA isolation, the cells were detached with a rubber policeman, centrifuged and suspended in RL buffer (EURx, Gdansk, Poland). Afterward the total RNA was extracted using Universal RNA purification Kit (EURx, Gdansk, Poland), according to the manufacturer’s protocol. MMLV reverse transcriptase (Promega, Madison, WI, USA) was used for cDNA synthesis. The reverse transcription reaction was performed with ProFlex PCR System (Applied Biosystems, Foster City, CA, USA). The QuantStudio 7 Flex (Applied Biosystems, Foster City, CA, USA) was employed for the real-time qPCR. Blank qPCR Master Mix (EURx) and the following Taq-Man human probes (Applied Biosystems) were used: HIF1A (Hs00153153_m1), MYC-C (Hs00905030_m1), BCL2 (Hs00153350_m1), BAX (Hs001802669_m1), CCND1 (Hs00277039_m1) and GAPDH (Hs99999905_m1). The obtained data were normalized against GAPDH transcript as a reference gene and levels of RNA expression were determined with the 2^−ΔΔ*C*t^ method.

### 2.9. Unsaturated Fatty Acid Content

C4-I cells were exposed to CA, Met and CA/Met for 24 h, washed with buffered PBS (Lonza) and collected. Previous to the unsaturated fatty acid assay, the organic extraction of total lipids from the cells was performed according to the manufacturer’s protocol using Chloroform-free Lipid Extraction Kit (Cell Biolabs Inc., San Diego, CA, USA). Then content of unsaturated fatty acids in cells was measured with sulfo-phospho-vanillin method using commercially available colorimetric kit (Cell Biolabs Inc.). Briefly, the samples were treated with 18M sulfuric acid and incubated for 10 min at 90 °C and then for 5 min were kept in 4 °C. Then vanillin reagent was added to each sample and the mixture was incubated for 15 min at 37 °C. The absorbance was measured at 550 nm using Bio-Tek microplate reader (the background was subtracted from signal).

### 2.10. MTT Assay

Cell proliferation was detected by MTT (3-[4,5-dimethylthiazol-2yl]-2,5-diphenyl tetrazolium bromide purchased from Sigma-Aldrich (Seelze, Germany). C4-I exponentially dividing cells (2.5 × 10^5^ cells/mL) were grown in 96-well microtiter plate (Sarstedt, Numbrecht, Germany) in serum-free medium. For each experiment, the positive controls (the untreated cells cultured in medium with addition of appropriate solvent) were prepared. After incubation with compounds, the medium was removed and replaced with the new one, containing 0.5% *w*/*v* MTT. The formazan crystals generated during incubation were dissolved in DMSO and absorbance was recorded at 570 nm (the reference wavelength was 630 nm), as described previously [18,25]. Microplate reader (Tecan, Salzburg, Austria) was used for absorbance measurement. The relative cellular growth was determined by a ratio of average absorbance in cells exposed to tested compounds versus the average absorbance in untreated cells.

### 2.11. LDH Assay

Lactate Dehydrogenase (LDH) leakage assay was used to detect the cytotoxicity of CA, Met and co-treatment in culture of C4-I cells. LDH is elevated in media following incubation of the cells with cytotoxic agents. For LDH test, the cells were seeded into 96-well plates (Sarstedt) and exposed to drugs as described in Section 2.11. The commercially available kit from Biolabo S.A. (France) was used to measure LDH leakage from cells exposed to the tested compounds. The LDH test was performed according to the manufacturer’s protocol and the LDH level in medium was quantified by an enzymatic reaction of the conversion of pyruvate and lactate with subsequent reduction of NAD^+^ to NADH. The concentration of generated NADH was measured at 340 nm using microplate reader (Tecan, Austria). The results were calculated as the percentage of LDH in the medium versus total LDH activity in the cells.

### 2.12. Reverse Transcription-Polymerase Chain Reaction (RT-PCR)

The cells were incubated as described in Section 2.7 and subsequently lysed. Initially, cell lysate was spun through the spin column eliminating the genomic DNA and then total RNA was purified with silica-membrane RNeasy spin columns (Qiagen, Hilden, Germany). Next, the isolated RNA was subjected to measurement using NanoDrop ND-1000 Spectrophotometer (Nano Drop Technologies, Wilmington, DE, USA). For cDNA synthesis 1ug of total RNA was used. Synthesis of cDNA was carried out using the oligodT starter and GoScript Reverse Transcriptase (Promega GmbH, Mannheim, Germany). The PCR reaction mixture was composed of 1.5 uL of cDNA, 0.2 mM dNTPs (Sigma Aldrich), 0.2 µM of each primer (Sigma Aldrich), 2.5 mM Magnesium Chloride (Sigma Aldrich), 1.25 U JumpStart™ Taq DNA Polymerase (Sigma Aldrich and 1× PCR buffer (Sigma Aldrich) in final volume 25 µL. Amplification of cDNA with the specific primers was performed in Research PTC-200 Thermal Cycler. Primers used were: SLC2A1 (Solute carrier family 2, facilitated glucose transporter member 1, GLUT1) forward (5-TTGGCTCCGGTATCGTCAAC-3), SLC2A1 reverse (5-GGTCCGGCCTTTAGTCTCAG-3), SLC2A3 (Solute carrier family 2, facilitated glucose transporter member 1, GLUT3) forward (5-GTGCACTGTCACTTTGCTCTG-3), SLC2A3 reverse (5-AACCTACTGTTTGAGGAGCCAG-3), HK2 (hexokinase 2) forward (5-TCACGGAGCTCAACCATGAC-3), HK2 reverse (5-CTGCAGTAGGGTGAGTGGTG-3), ALDOA (Aldolase A) forward (5-GAGAACACCGAGGAGAACCG-3), ALDOA reverse (5-TGGGTGGTAGTCTCGCCATT-3), ENO1 (enolase 1) forward (5-ATGGTGTCTCATCGTTCGGG-3), reverse (5-TGAGGAGCTGGTTGTACTTGG-3), PKM (pyruvate kinase M1/2) forward (5-GCCGCCTGGACATTGATTC-3), reverse (5-CCATGAGAGAAGTTCAGACGAG-3), PDK1 (pyruvate dehydrogenase 1) forward (5-CGGATCAGAAACCGACACAA-3), reverse (5-AGATGGACTTCCTTTGCCTTTTC-3), PFKFB4 (6-Phosphofructo-2-Kinase/Fructose-2,6-Biphosphatase 4) forward (5-GGGATGGCGTCCCCACGGG-3), PFKFB4 reverse (5-CGCTCTCCGTTCTCGGGTG-3), LDHA (Lactate Dehydrogenase A) forward (5-CTGTTCCACTTAAGGCCCCTC-3), LDHA reverse (5-CCAGCCTTTCCCCCATTAGG-3), HPRT1 (Hypoxanthine-Guanine Phosphoribosyltransferase) forward (5-TGGCGTCGTGATTAGTGATG-3), HPRT1 reverse (5-TATCCAACACTTCGTGGGGT-3). The condition for PCR reaction was as following; 5 min of denaturation at 95 °C, next 30 s annealing at 58 °C and 30 s extension at 72 °C. The final extension was performed at 72 °C for 10 min. Amplification for 30 cycles was applied for all PCR products with subsequent visualization on the agarose gel (1.5% *w*/*v*). HPRT gene was used as housekeeping gene for normalization purpose.

### 2.13. Statistical Analysis

The data were expressed as mean ± SD. The statistical significance of the obtained raw data was analyzed using one-way ANOVA and followed by Duncan post-hoc test (*p* values < 0.05, *p* < 0.01, *p* < 0.001) using the commercially available packages Statistica PL v.10 (StatSoft, Tulsa, OK, USA). 

## 3. Results

### 3.1. CA Treatment Impairs Glutaminolysis by Downregulation of Glutaminase (GLS) and Decreases NADPH Level in C-4I Cells

**Figure 1:**
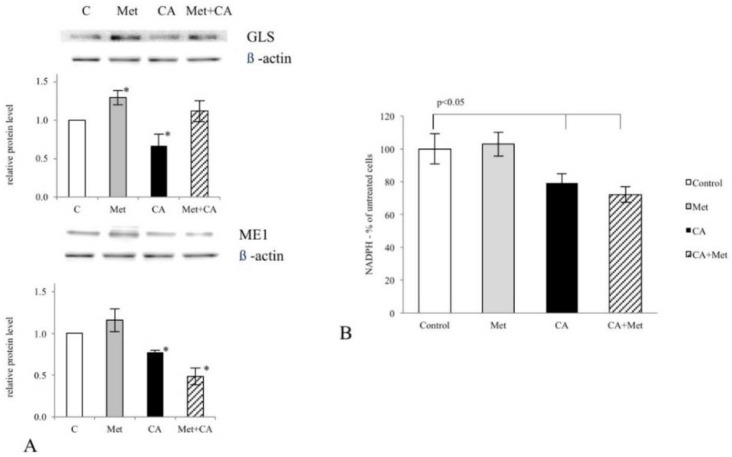
The effect of Caffeic Acid (CA) and Metformin (Met) on Glutaminase (GLS) and Malic Enzyme 1 (ME1) expression and NADPH level in C-4I cells. The cells were treated with CA (100 μM) and/or Met (10 mM) for 24 h in serum-free medium. The exposition of cells to CA decreased GLS and ME1 expression (**A**) (* *p* < 0.05 vs. untreated control) Protein levels of GLS and ME1 were analyzed by Western blot, as described in Materials and Methods. CA caused decrease of NADPH level in C-4I cells, as measured spectrophotometrically (**B**). Data presented are representative of those obtained in three separate experiments.

The effect of Met and CA on glutamine anaplerosis in C-4I cells was evaluated by estimating capability of drugs to influence the expression of GLS. CA treatment at a concentration of 100 μM for 24 h decreased GLS protein amount, while Met at 10 mM caused minor upregulation of the enzyme (Figure 1A, *p* < 0.05 vs. untreated control). CA decreased the expression of Malic Enzyme 1 (ME1) protein amount and thus we determined whether the incubation of cells with the drug may influence the intracellular NADPH level. The obtained results indicated that CA alone and co-treated with Met caused significant reduction of NADPH content in C-4I cells (Figure 1B, *p* < 0.05 vs. untreated control).

### 3.2. CA Regulates TCA Cycle Supply via Pyruvate Dehydrogenase Complex (PDH), Induces Mitochondrial ROS Generation and Evokes Apoptosis in C-4I Cells

**Figure 2:**
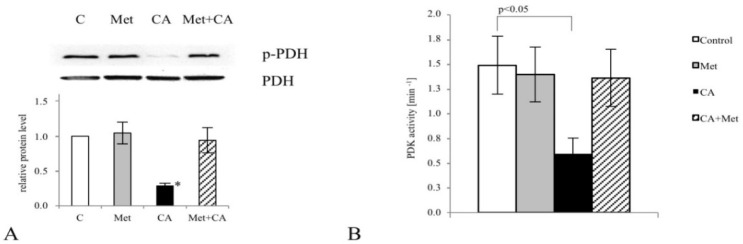
The effect of Caffeic Acid (CA) and Metformin (Met) on pyruvate decarboxylation via Pyruvate Dehydrogenase Complex (PDH) in C-4I cells. The cells were exposed in serum-free medium to CA (100 μM) and/or Met (10 mM) for 24 h. Protein level was assayed by Western blot as described in Materials and Methods and followed by chemiluminescent detection. β-actin was used as the protein loading control (**A**). Note that CA, unlike Met and Met/CA, caused PDH-E1α phosphorylation on S^293^ residue ((**A**), * *p* < 0.05 vs. untreated cells) and inhibited Pyruvate Dehydrogenase Kinase (PDK) activity, as measured spectrophotometrically (**B**). The suppression of PDK resulted in restoration of PDH complex and enhanced pyruvate flux to mitochondria. Data presented are representative of those obtained in three separate experiments.

As shown in Figure 2A, CA promoted the process of oxidative decarboxylation of pyruvate in C-4I cells by dephosphorylation of PDH complex at S^293^ residue (*p* < 0.05 vs. untreated control). To explore the mechanism of the PDH inhibition, we tested whether the drug affects the activity of Pyruvate Dehydrogenase Kinase (PDK). PDK phosphorylates and thereby inactivates PDH complex. Exposition of cells to CA at a concentration of 100 µM caused inhibition of PDK activity, as demonstrated in Figure 2B, (*p* < 0.05 vs. untreated control).

**Figure 3:**
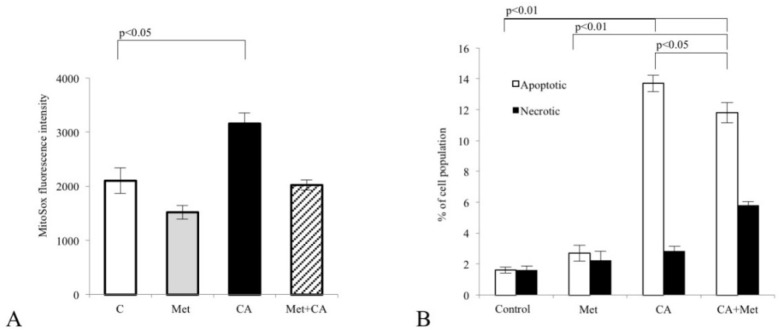
The effect of Caffeic Acid (CA) on mitochondrial ROS formation and apoptosis in C-4I cells. The treatment of cells with CA for 24 h caused oxidative stress in mitochondria, as measured using MitoSox Red (**A**). The enhanced ROS generation was followed by cell death due to apoptosis (**B**) Apoptosis/necrosis was determined by flow cytometry followed by Annexin V/EthD-III double staining, as described in Materials and Methods. The number of apoptotic/necrotic cells was presented as a percentage of total cells. CA was used at 100 µM and Metformin (Met) at 10 mM and the cells were incubated for 24 h in serum-free media. Experiments were repeated three times with similar results and presented as mean values ± SD.

To explore whether C-4I cells exposed to CA for 24 h become more vulnerable to oxidative stress, MitoSox staining followed by cytometry analysis was used. Single treatment of the cells with CA significantly increased ROS level in mitochondria, compared to untreated control ((Figure 3A; *p* < 0.05), while Met itself caused no statistically significant effect (*p* < 0.05 vs. untreated control). Using flow cytometry, we also determined that apoptosis was the supreme way of death of cells incubated with CA. The percentage of apoptotic cells after CA and Met/CA treatment was significantly higher than in population of untreated or Met-treated cells, as presented in Figure 3B (*p* < 0.01 for CA and Met/CA vs. untreated control, *p* < 0.01 for Met/CA vs. Met). The number of apoptotic cells in population after CA treatment was significantly higher than corresponding amount in population of Met/CA-treated cells (*p* < 0.05 for CA vs. Met/CA).

### 3.3. CA Activates AMPK and Attenuates Lipid De Novo Biosynthesis in C-4I Cells by Suppression of Regulatory Enzymes of the Pathway

**Figure 4:**
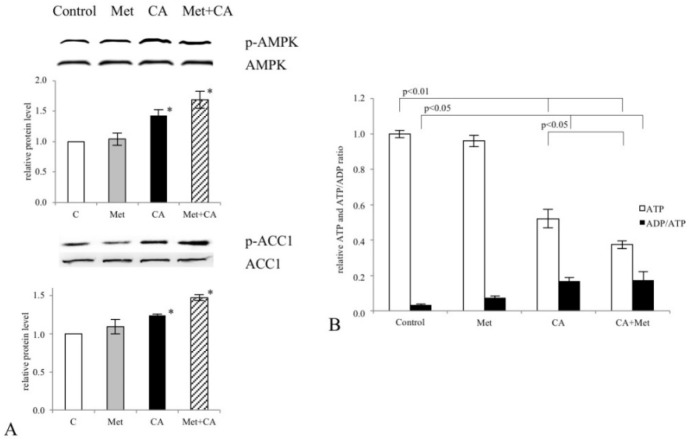
Activation of AMPK by Caffeic Acid (CA) and co-treatment in C-4I cells. Human cervical carcinoma C-4I cells were grown in serum-free medium and were incubated with Metformin (Met, 10 mM), CA (100 μM) or both compounds for 24 h. The amounts of enzyme protein in cell lysates were measured by Western blot analysis and followed by chemiluminescent detection (β-actin was used as the protein loading control, the details described in Material and Methods). Note that exposition of cells to CA and Met/CA increased the phosphorylation of AMPK on T^172^ residue (**A**). AMPK downstream protein, Acetyl-CoA Carboxylase 1 (ACC1), was phosphorylated at S^78,80^ due to CA and Met/CA treatment. The phosphorylation of ACC1 resulted in the decrease of its activity. CA alone and Met/CA significantly decreased ATP content in cancer cells, as measured with spectrophotometric assay (**B**) Data shown here are from a representative experiment repeated three times with similar results, band intensities were quantified by densitometry analysis, * *p* < 0.05 vs. untreated cells (**A**) or presented as mean values ± SD for ATP and ADP/ATP (**B**).

We explored the ability of CA, Met and combined treatment of compounds to activate AMPK in C-4I cells after 24 h of incubation. Western blot analysis was employed to find out whether tested drugs may affect the phosphorylation of the enzyme on the T^172^ residue. Results showed that CA and Met/CA activated AMPK while Met failed to phosphorylate the enzyme (Figure 4A, *p* < 0.05 vs. untreated control). As presented in Figure 4B, the treatment of cervical carcinoma cells with CA and Met/CA significantly reduced intracellular ATP level (*p* < 0.01 vs. untreated control for CA and *p* < 0.01 vs. untreated control for Met/CA). CA and Met/CA caused phosphorylation of AMPK downstream protein Acetyl-CoA Carboxylase 1 (ACC1) and deactivated the enzyme by its phosphorylation at S^78,80^ (Figure 4A, *p* < 0.05 vs. untreated control).

**Figure 5:**
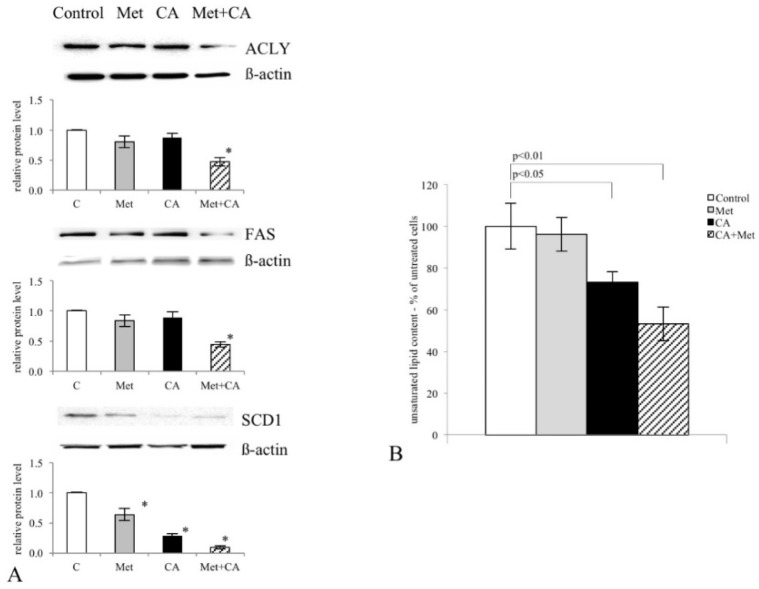
Inhibition of ATP Citrate Lyase (ACLY), Fatty Acid Synthase (FAS) and Stearoyl-CoA Desaturase-1 (SCD1) expression caused by Caffeic Acid (CA), Metformin (Met) and co-treatment and following decrease in total unsaturated FA content in C-4I cells. Immunoblot analysis (the details described in Materials and Methods) revealing that co-treatment of cells with CA and Met for 24 h restrained the expression of regulatory enzymes of lipid de novo synthesis ACLY and FAS. Note that CA, Met and CA/Met downregulated SCD1, a key enzyme catalyzing the formation of double bond in FA ((**A**),* *p* < 0.05 vs. untreated cells), which was followed by decrease of the level of total unsaturated FA in C-4I cells, as measured using spectrophotometric assay ((**B**), mean values ± SD were presented, *n* = 3).

Next, we investigated whether Met and CA may affect the process of lipid biosynthesis. Therefore, we tested if the compounds may exert an effect on the expression of regulatory enzymes controlling FA de novo synthesis, ATP Citrate Lyase (ACLY) and Fatty Acid Synthase (FAS). We found that both drugs inhibited the expression of ACLY and FAS, but the greatest effect was detected after co-treatment, as shown in Figure 5A (*p* < 0.05 vs. untreated control). Additionally, the exposition of cells to CA and Met/CA reduced Stearoyl-CoA Desaturase-1 (SCD1) protein amount (Figure 5A), which was followed by a significant decrease in unsaturated FA content in C-4I cells (Figure 5B, *p* < 0.05 for CA vs. untreated control, *p* < 0.01 for Met/CA vs. untreated control)

### 3.4. Met Decreases HIF-1α Protein Stability under Hypoxia and Inhibits the Expression of HIF-1α Downstream Efectors in HTB-35 Cells

**Figure 6:**
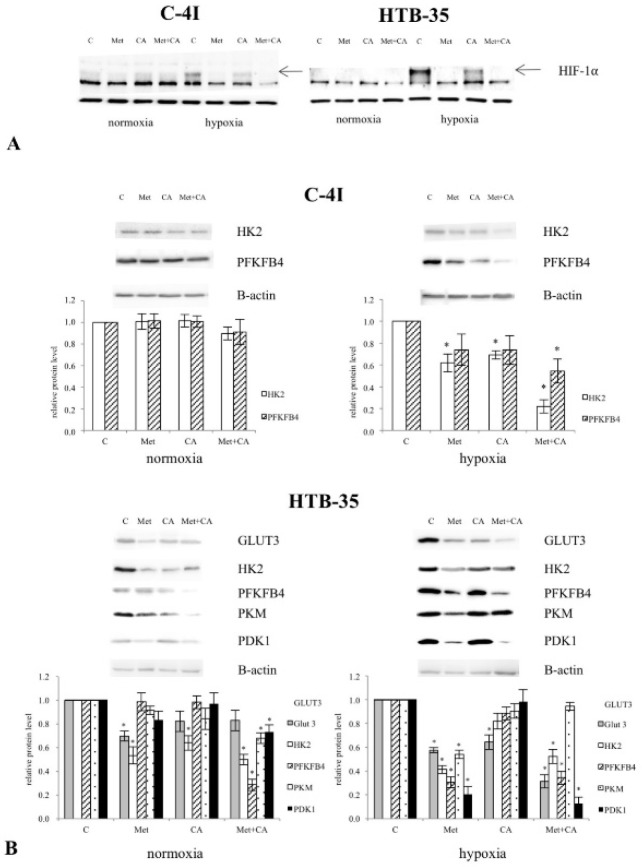
The effect of Caffeic Acid (CA) and Metformin (Met) on HIF-1α degradation (**A**) and expression of glycolytic regulatory proteins (**B**) in C-4I and HTB-35 cells. The cells were treated for 24 h with CA (100 μM) and/or Met (10 mM) either in normoxia (21% O_2_ level) or in hypoxia (5% O_2_ level). Protein levels of HIF-1α was analyzed by Western blot and shown in Figure 6A. Note that hypoxia elevated the amount of HIF-1α protein in HTB-35 cells. Met caused the greatest decrease of HIF-1α protein level in HTB-35 cells (**A**). Representative immunoblots show that treatment of HTB-35 cells with Met during hypoxia decreased protein amounts for Glucose transporter 3 (*GLUT3*), Hexokinase 2 (*HK2*), 6-Phosphofructo-2-Kinase/Fructose-2,6-Biphosphatase 4 (*PFKFB4*), Pyruvate Kinase (*PKM*) and Pyruvate dehydrogenase kinase 1 (*PDK 1*) ((**B**), * *p* < 0.05 vs. untreated cells) Analysis of protein level was performed with Western blot (30 μg of protein was loaded on the polyacrylamide gel, the procedure was described in Material and Methods, β-actin was used as a reference). Experiments were repeated three times with similar results and presented as mean values ± SD.

To gain more insight into the overall effect of CA and Met on metabolic reprogramming of HTB-35 cells, here we aimed to determine, whether Met may regulate the key points of glycolytic pathway. In the current study we used C-4I cells and HTB-35 cells for comparative reasons. As HIF-1α plays a central role in the regulation of glycolytic phenotype of tumor cells [27], we performed Western blot analysis to test the effect of drugs on HIF-1α under hypoxic and normoxic conditions. We found that in HTB-35 cells HIF-1α protein was induced under hypoxia, while in normoxic conditions the protein was not detected (Figure 6A). In C-4I cells hypoxia induced HIF-1α, but we found that the protein was expressed in normoxia as well. As shown on immunoblots, the exposition of HTB-35 cells the cells to 10 mM Met suppressed HIF-1α. CA treatment at 100 μM for 24 h also inhibited HIF-1α (*p* < 0.05 vs. control).

To assess whether Met regulates the expression of HIF-1α-dependent proteins in cervical cancer cells we performed Western blot analysis. As demonstrated on immunoblots, in hypoxic HTB-35 cells Met decreased transcript for Glucose transporter 3 (*GLUT3*), Hexokinase 2 (*HK2*), 6-Phosphofructo-2-Kinase/Fructose-2,6-Biphosphatase 4 (*PFKFB4*), Pyruvate Kinase (*PKM*) and Pyruvate dehydrogenase kinase 1 (*PDK 1*) under hypoxic conditions. CA exerted weaker effect on expression of glycolytic regulatory genes in HTB-35 cells (Figure 6B). We detected minor changes in the protein level for glycolytic enzymes in C4-I cells under CA, Met and co-treatment.

### 3.5. Met Evokes Apoptosis and Downregulates BCL-2, CCND1 and c-Myc in HTB-35 Cells

**Figure 7:**
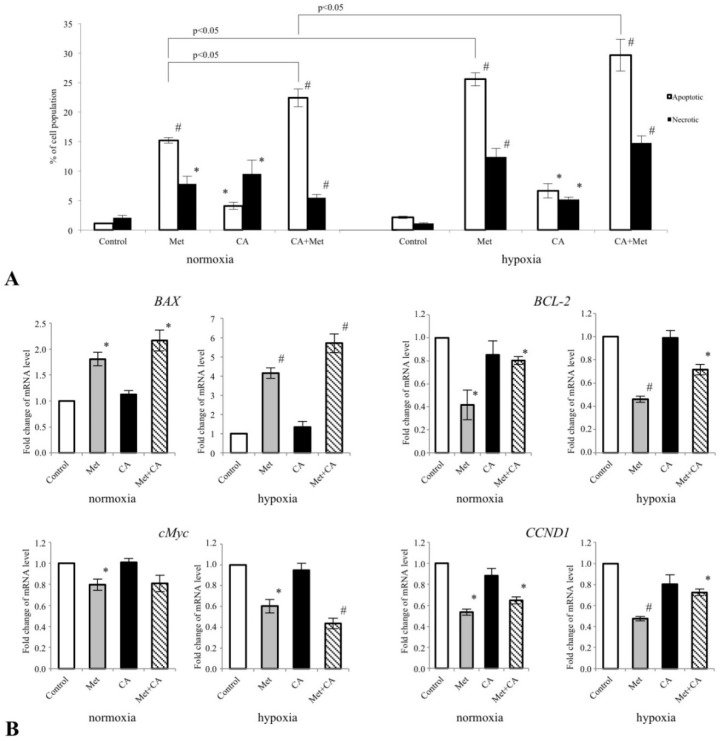
The effect of Metformin (Met) and Caffeic Acid (CA) on apoptosis and proliferation in HTB-35 cells. The cells were incubated either in normoxia (21% O2 level) or in hypoxia (5% O2 level) with CA (100 μM), Met (10 mM) or both compounds for 24 h. Met promoted cell death due to apoptosis ((**A**), *p* < 0.05 vs. control) and inhibited the expression of B-cell lymphoma 2 (*BCL-2*), Cyclin D1 (*CCND1*) and Proto-Oncogene C-Myc (*c-Myc*) genes in HTB-35 cells (**B**). Data shown here are representative of three experiments performed with similar results. (**A**) Apoptosis was determined by flow cytometry (Annexin V/EthD-III double staining), (**B**) the expression of mRNA under normoxic and hypoxic conditions was analyzed with qPCR (the data were normalized against reference gene Glyceraldehyde 3-phosphate dehydrogenase (*GAPDH*) transcript; the 2^−ΔΔ*C*t^ method was used for determination of RNA level; * *p* < 0.05 and ^#^ *p* < 0.01 vs. control).

We analyzed, if the exposition of the cells to drugs may result in facilitated apoptosis in HTB-35 cells. As presented in Figure 7A, the incubation of the cells in normoxia with Met at concentration of 10 mM caused tumor cell death due to apoptosis, as measured using flow cytometry (*p* < 0.01 vs. untreated control). Co-treatment caused the greatest increase of apoptotic cells under normoxia and hypoxia (*p* < 0.01 vs. untreated control for normoxic conditions, *p* < 0.05 vs. untreated control for hypoxic conditions). The percent of apoptotic cells in population following Met treatment in hypoxic conditions was higher than apoptosis detected in normoxia (*p* < 0.05 Met in normoxia vs. Met in hypoxia).

**Figure 8:**
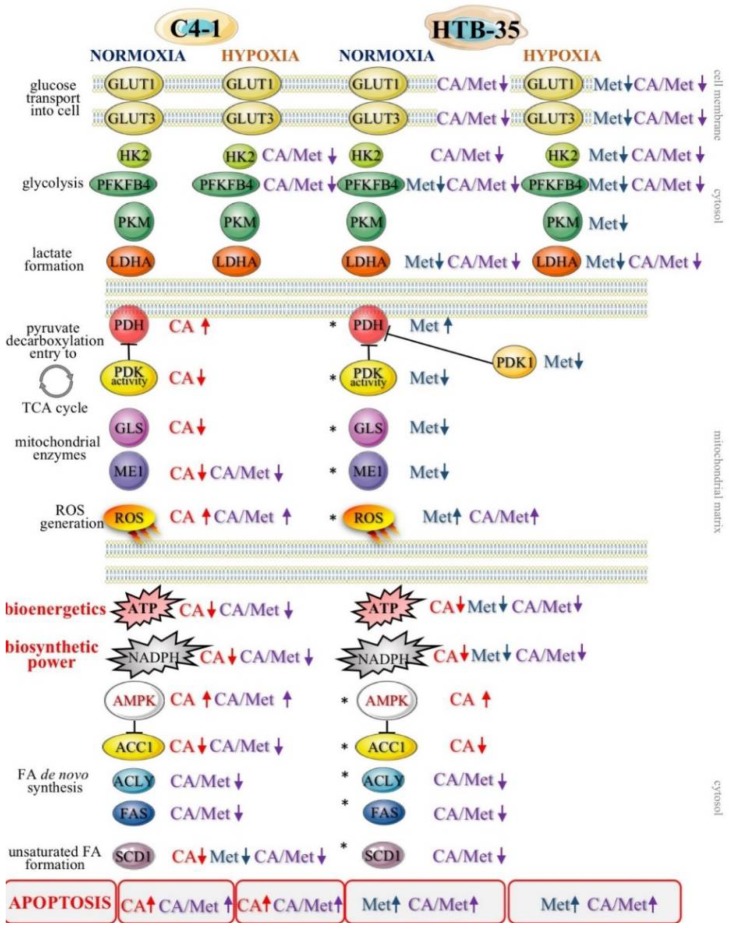
CA, Met and co-treatment regulate metabolism of human cervical carcinoma C-4I and HTB-35/SiHa cells under normoxia and hypoxia. Schematic overview of the cellular effects of the tested compounds includes aerobic glycolysis and mitochondrial bioenergetics/biosynthetic processes (the supplementation of TCA cycle by decarboxylation of pyruvate via PDH complex, glutaminolysis of glutamate via GLS, NAPPH formation via ME1) and the synthesis of unsaturated FA. The main actions of CA, Met and CA/Met on regulatory proteins and processes were marked with arrows (↑ activation, ↓ inhibition). The previous results [18] denoted by the asterisk (*).

As the proteins of Bcl-2 gene family are involved in the apoptotic signaling, we determined if CA and Met affect the expression of genes encoding *BAX* and *BCL-2* proteins. We used real-time RT–PCR to analyze the effects at the mRNA level. Apoptosis regulator BAX by interaction with the mitochondrial voltage-dependent anion channel (VDAC) facilitates to the loss in membrane potential thereby enabling the release of cytochrome c from mitochondria and following apoptotic death. On the other hand, Bcl-2 (B-cell lymphoma 2) protein promotes tumor cell survival by inhibiting the actions of pro-apoptotic proteins. We determined, that under hypoxic and normoxic conditions Met increased the expression of pro-apoptotic *BAX* gene and downregulated anti-apoptotic *BCL-2* gene (Figure 7B). Met also suppressed the expression of cyclin-D1 (*CCND1*). Cyclin-D1 plays the key role in regulation of the cell cycle progression of cancer cells and in HTB-35 cells exposed to Met the two-fold decrease of *CCND1* transcript level was found under normoxia (*p* < 0.05 vs. untreated control) and under hypoxia as well (Figure 8B, *p* < 0.05 vs. untreated control). Given that c-Myc transcription factor controls the expression of glycolytic genes and regulates proliferation of tumor cells [28], we examined the expression of *c-Myc* in HTB-35 cells. The results showed that Met reduced the mRNA for *c-Myc* (*p* < 0.05 vs. control) in HTB-35 cells exposed to hypoxia and normoxia (Figure 7B).

## 4. Discussion

In prior studies we showed that small molecules Met and CA may influence mitochondrial oxidative metabolism in metastatic human cervical squamous carcinoma cell lines, HTB-35 [18] and HTB-34 [25]. As the TCA cycle plays a key role in determining of “flexible” metabolic phenotype of neoplastic cells [1,22,24], here we aimed to gain more insight into the effect of CA and Met on cellular bioenergetics and biosynthetic capacity of C4-I epithelial cells derived from in situ cervical tumor. In particular, increased glutaminolysis via GLS effectively replenishes TCA cycle pool for citrate synthesis [22,28]. Our results show that CA at 100 μM limits TCA cycle supplementation with glutamine by decreasing GLS expression in C-4I cells. What is more, in tumor cells glutamine entry to TCA cycle improves not only carbon supply for macromolecules build-up, it also may replenish pool of cellular NADPH, since the conversion of malate to pyruvate catalyzed by ME1 is accompanied by a reduction of NADP^+^ molecule. In cancers, ME1 is active enough to provide sufficient level of reductive power for lipid biosynthesis [29] and de novo synthesis of cellular phospholipids may be sustained even if the glycolytic generation of NADPH is shut down [3]. During oxidative stress NADPH play key role in reinforcing the anti-oxidative protection of tumor cell by the reduction of glutathione molecules [2]. In the current study CA treatment of C-4I cells causes downregulation of ME1 together with a significant decrease of NADPH level. Therefore, we may presume that the impaired generation of NADPH results not only in alleviation of biosynthesis, but also in the reduced ability of tumor cells to combat ROS generated in oxidative mitochondrial metabolic pathways.

What is more, CA treatment of C-4I cells causes the restoration of PDH “bottleneck”, which reduces the flux of pyruvate to lactate and additionally disturbs the generation of NADPH and ATP in cytosol. As a result, pyruvate enters mitochondrial oxidative metabolism via PDH complex [6]. In tumor cells, PDH plays a key role in determining the fate of glucose. A specific PDH kinase (PDK) phosphorylates and inactivates PDH [2,30]. In culture of HeLa cervical tumor cells, PDK inhibitor dichloroacetate (DCA) was recently demonstrated to reduce glycolysis by activating PDH [31]. The similar effect of DCA was shown in glioblastoma cell line [32]. The present data suggest that CA may be another potent PDK inhibitor. In C-4I cells exposed to CA the enhanced fueling of TCA cycle with pyruvate leads to induction of intolerable oxidative stress and apoptosis (Appendix A). The similar effect we observed in cervical cancer HTB-34 cells treated with CA, as previously reported [25]. By contrast to C4-I cells, in invasive HTB-35 line Met at 10 mM was shown to activate oxidative decarboxylation of pyruvate, while CA had no significant effect [18]. The present study provides the possible explanation for such action of the drugs, since while in C4-I cells CA affects the activity of PDK, Met in HTB-35 cells targets the expression of gene encoding *PDK-1.* PDK-1 is a isoenzyme specific to cancer cells and its expression is controlled by HIF1α [29]. Interestingly, under hypoxic conditions the downregulation of *PDK-1* caused by Met is even greater than during normoxia. Therefore, CA activates PDH in epithelial C-4I cells and Met in aggressive HTB-35 cells. These findings imply that both drugs may precisely regulate PDH checkpoint in cervical tumor cell lines and the mechanism of action depends on the particular metabolic phenotype of the cells.

Our data show that CA and CA/Met treatments reduce intracellular ATP level in C-4I cells and causes energetic stress followed by AMPK activation. Several reports have suggested that once activated AMPK may act as a potent metabolic tumor suppressor [33]. However, it was also found, that AMPK activation and following inhibition of ACC1 may prevent NADPH consumption and promote survival of cancer cells, as reported by Park et al. [34] and Jeon et al. [35]. Our data suggest that in C-4I cells in conditions of limited TCA cycle anaplerosis/suppressed generation of reductive power for biosynthesis/disturbed cellular bioenergetics the activation of AMPK may result rather in anti-proliferative than pro-survival effect. Phytochemicals-rich strawberry extract stimulated AMPK and inhibited regulatory proteins of FA and cholesterol synthesis in Hep G2 cells [36]. It was demonstrated in colorectal carcinoma cells and hepatocellular carcinoma cell line that CA derivatives and Met may suppress tumor cells growth by inhibition of lipid de novo synthesis involving AMPK signaling [9,37,38,39]. Sánchez-Martínez et al. reported that activation of AMPK counteracts the induction of EMT phenotype triggered by the ACSL/SCD signaling network [40]. Interestingly, here the concerted action of the drugs causes the greatest inhibition of FA biosynthesis as well as cells’ proliferation (Appendix A). The co-treatment affected HTB-35 and HTB-34 cells in the similar way, thus we may presume that the regulatory points of FA de novo synthesis may be molecular targets for the drugs. Additionally to our previous results, here we demonstrated that the exposition of cervical cancer cells to double treatment impaired FA generation via AMPK/ACC1 axis [18,25]. Most notably, in the current study the exposition of the cells to CA, Met and CA/Met causes almost complete downregulation of SCD1, the enzyme that controls the key step in the synthesis of unsaturated FA. Considering the essential role of unsaturated FA in cancer cell metabolom, the total inhibition of unsaturated FA generation under co-treatment may suppress numerous intracellular processes, such as synthesis of tumor cell membranes. However, the obtained data are not enough to state that the activation of AMPK and following impairment of lipid synthesis is a conclusive mechanism restraining proliferation of cervical cancer cells. We will continue the study in order to elucidate mechanisms underlining this finding.

Likewise, it should be stressed that modulating AMPK in cervical cells may lead to various outcomes as a result. Thus, understanding the context-dependent effects of AMPK activation in cervical tumors may be critical to define precisely what kind of manipulation can be therapeutically beneficial. What is more, several molecular pathways by which AMPK mediate anticancer effects have been previously recognized. Liver Kinase B1 (LKB1)/AMPK/mTOR signaling is thought to be the fundamental mechanism behind the suppression of tumor cells proliferation. It was demonstrated, using biopsies of cervical tumor tissues, that somatic mutations may occur in LKB1 gene in humans, thereby hampering its function [41,42]. In this case the classical activators of LKB1/AMPK axis might be inefficient. However, it was also shown, that at the same time LKB1-deficient tumor cells are more sensitive to energy stress, which provides some opportunities for targeted therapy using small molecules that disrupt intracellular energy balance [5,15,41]. In line with this finding, CA in epithelial C4-I cells and Met in motile HTB-35 cells, by targeting energy homeostasis and reducing intermediates for biosynthesis, may be used for effective shut down of cancer cells growth. Our previous [18] and the current study have revealed that while CA was more active than Met in C4-I cells, the latter drug caused massive cell death in HTB-35 cell line (Figure 8).

Here, we tried to recognize the factors related to different cellular effects of the compounds. Our latest study demonstrated that both cell lines display different epithelial/mesenchymal gene expression profile [24,42], which may account for their susceptibility to the drugs. It was established that the activation of glycolysis a prevalent energy source for migrating cells, which undergo EMT to improve survival in a harsh environment and facilitate metastasis [3]. As the glycolytic phenotype of tumor cell is triggered mainly by HIF-1α master regulator, we sought to find out, whether the drugs may exert its effect on metabolic reprogramming of HTB-35 cell via HIF-1α and its downstream proteins. The overexpression of HIF-1α efficiently supports metabolic reprogramming of tumors [26,43]. Our study showed that Met inhibited hypoxia-induced upregulation of HIF-1α and its downstream proteins, which was demonstrated using Western blot analysis. Met effectively restrained the glycolytic phenotype of HTB-35 cells by downregulation of glucose transporters *GLUT1* and *GLUT3*, by suppressing of *HK2,* the limiting enzyme of glycolysis, and by inhibiting regulatory enzymes of the pathway, *PFKFB4*, *PKM* and *LDH* (Figure S1). Under hypoxic conditions inside some tumors, the activation of HIF-1α restricts glucose and glutamine entry into the TCA cycle by suppressing of PDH and α-KG dehydrogenase (α-KGDH) activities. Therefore, the triggering of HIF-1α-dependent program promotes survival of neoplastic cells by decreasing mitochondrial metabolism, adjusting TCA flux and minimalizing of oxidative stress within mitochondria [44]. Our study shows that in HTB-35 cells Met may counteract these metabolic alterations by decreasing *PDK1* gene expression, which leads to augmented funneling of TCA cycle and results in impairment of glycolysis. It should be clearly recognized that at the same time the effect of CA on glycolysis in HTB-35 cells was minor.

Recent studies have reported that enhanced expression of the oncogene c-Myc is involved in the formation of cervical cancer [28]. Additionally, the upregulated c-Myc may collaborate with HIF in the induction of glycolytic phenotype of tumor cell, increased glucose consumption, decreased oxidative phosphorylation and accompanying lactate production [45]. Therefore, we presume that the downregulation of *c-Myc* in HTB-35 cells treated with Met and CA/Met may additionally hamper glycolytic phenotype of the cells. The treatment of HTB-35 cells with Met caused the suppression of cyclin D1 gene (*CCND1),* which in most tumor cells is positively regulated by c-Myc. These effects, together with the upregulation of pro-apoptotic *BAX,* may facilitate apoptosis in HTB-35 cells.

In C4-I cells CA suppressed c-Myc (Figure S2), GLS expression, and thereby glutamine supply to TCA cycle. The cells starving glutamine are more sensitive to first apoptosis signal (FAS) receptor ligands and more vulnerable to death via FAS-dependent extrinsic apoptotic pathway [46]. However, we did not investigate comprehensively, whether C4-I cells are more susceptible to apoptosis elicited via external factors following incubation with CA. We only aimed to point out that regulation of metabolic reprogramming of cervical cancer cells may result in decreased cell survival. As mentioned, the increased ROS level may trigger cell death via apoptotic intrinsic pathway. It has been reported before that CA may evoke mitochondrial apoptosis in HeLa cervical tumor cells via inhibiting BCL-2 activity, release of cytochrome-c from mitochondria and activation of caspase 3 [37,47]. In the current study we have found that in C4-I cells CA upregulates pro-apoptotic promoter BAX protein (Figure S2). Activation of BAX may lead to disruption of mitochondrial membrane potential and to a cascade of events leading to apoptotic cell death. In the present work we have not determined, whether the exposition of C4-I cells to CA may trigger such intracellular events. However, our data shows that CA suppresses the expression of “primer oncogene” c-Myc and its downstream gene *CCND1*. Interestingly, in C4-I cells Western blot analysis revealed the elevated expression of HIF-1α protein even during normoxia and we assume that the process of induction of HIF-1α-dependent genes may be altered. This finding provides possible explanation for a minor effect of CA and Met on glycolytic pathway in C4-I cells.

In our previous and present experimental models, CA was used at a relatively high concentration (100 μM) whereas the micromolar concentration of CA is rather transient following oral application in humans [6]. Met was used at a concentration of 10 mM, while plasma concentration of the drug after single oral administration in diabetic patients is around 200-fold less [48]. However, it has been demonstrated that Met may accumulate in mitochondrial matrix even 1000-fold more than average level measured in human blood [49]. We can hypothesize that the location of cervical cancer within the body provides the opportunity to use the topical formulation containing the drugs at adequate concentration to evoke the anti-tumor effect. The topical ointment formulation of catechins was approved by The United States Food and Drug Administration for virucidal use in humans, and the relevant preventive effect of polyphenols against HPV infection has been demonstrated before [50]. Additionally, CA and Met can synergize with targeted therapies to enhance therapeutic response in specific tumor types [17,18]. Karthikeyan et al. reported that ferulic acid improved the outcome of radiation therapy against human cervical carcinoma cells [51]. CA pre-treatment sensitized ovarian cancer cells to the cytotoxic activity of cisplatin [16]. Met enhanced the action of tamoxifen in breast cancer [17]. As discussed elsewhere, CA and Met are absorbed and metabolized by cancer cells [6,11] and are not toxic to normal cells [18] However, our data show that Met may to a small extent reverse CA-dependent GLS inhibition in C4-I cell line. The mechanism of such an effect is not clear, since our current data showed that c-Myc, the oncogene that controls GLS expression, was not activated by Met. The underlined mechanism is probably more complex, and we cannot elucidate it based only on existing observations. However, this study may provide more insights into the existing results on the action of the drugs in cervical cancer cells and the treatment using tested drugs in vivo should be carefully considered, especially against the tumor cells that are glutamine-addicted. We may also presume, that the high amounts of nutrients in cell culture medium in present experiments, especially glutamine, might have extremely supported cancer cell growth and the effect of compounds in vivo may be different comparing to in vitro conditions [41,48].

## 5. Conclusions

The present study provides evidence that CA and Met induce energetic stress and impair the generation of reductive power for biosynthesis and anti-oxidative protection of cervical cancer cells. Additionally, each compound triggers specific mechanisms in the cells expressing epithelial and mesenchymal phenotypes. CA funnels carbon flux towards the TCA cycle which results in upregulation of ROS production and decreased survival of epithelial C4-I cells. Met acts as a glycolytic inhibitor under normoxic and hypoxic conditions via the downregulation of HIF1α and *c-Myc* in aggressive HTB-35 cells which undergo EMT. The use of both compounds may be therapeutically beneficial against invasive cervical tumor cells such as HTB-35 line, while the effect of co-treatment on mitochondrial regulatory enzymes in C4-I cells is minor. Application of CA and Met in humans may provide a promising approach in the prevention of cervical cancer progression regarding specific phenotypes of cells and in respect of metabolic and genetic profiles of tumor cells.

## Figures and Tables

**Figure A1:**
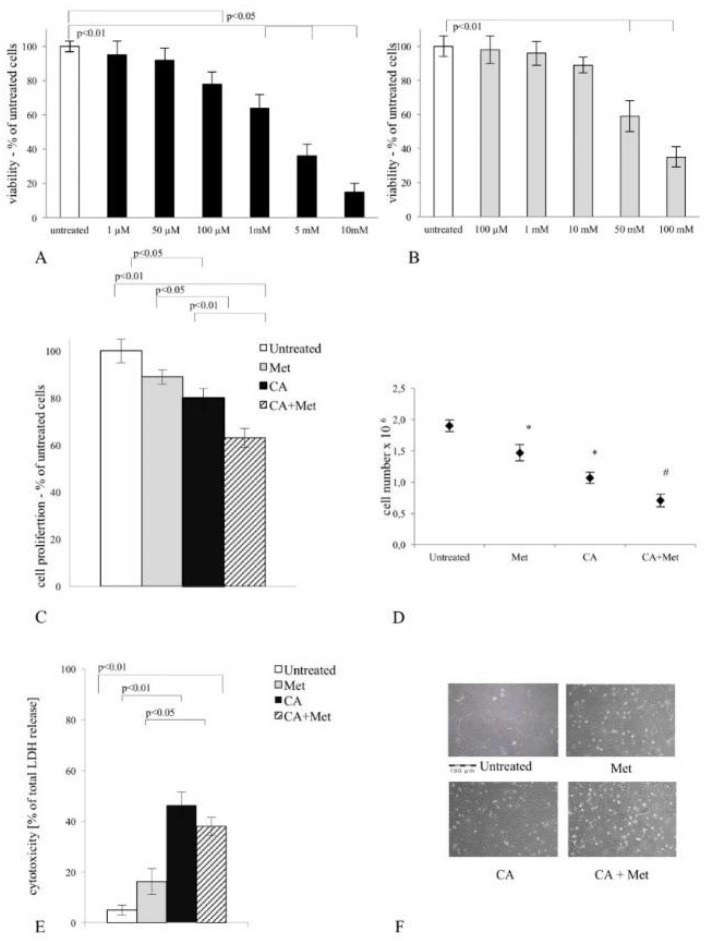
Inhibitory effect of CA treatment on proliferation and growth of C-4I cells. Cervical carcinoma cells were cultured in serum-free medium and exposed to Met at concentration ranged from 100 μM to 100 mM for 24 h. The anti-proliferative activity of CA was assessed using MTT colorimetric assay (**A**). The untreated cells were controls. The effect of Met was tested and measured in the same experimental conditions (**B**). After incubation of C4-I cells with mixture of 100 μM of CA and 10 mM of Met, the proliferation of cells was inhibited compared to untreated cells, (*p* < 0.01) (**C**). The decrease of the number of cells after Met/CA treatment was also assessed (using automatic cell counter, * *p* < 0.05 and * < 0.01 vs. control) (**D**). LDH colorimetric assay revealed that CA and Met/CA were cytotoxic to C-4I cells (*p* < 0.01 vs. untreated cells) (**E**) and aggravated the morphology of monolayers, as shown in phase contrast microphotographs (**F**).

## Supplementary Materials

Supplementary materials can be found at http://www.mdpi.com/2072-6643/10/7/841/s1. Figure S1: Met downregulates transcripts for glycolytic enzymes in HTB-35 cells. The cells were treated for 24 h with CA (100 μM) and/or Met (10 mM) either in normoxia (21% O2 level) or in hypoxia (5% O2 level). RT-PCR analysis was used and HPRT1 was a reference gene. Experiments were repeated three times with similar results and presented as mean values ± SD, Figure S2: The effect of Metformin (Met) and Caffeic Acid (CA) on expression of BAX, BCL-2, c-Myc and CCND1 genes in C-4I cells. The cells were incubated either in normoxia (21% O2 level) or in hypoxia (5% O2 level) with CA (100 μM), Met (10 mM) or both compounds for 24 h. CA inhibited the expression of CCND1 and c-Myc and CA/Met suppressed BCL-2 gene in HTB-35 cells (B). Data shown here are representative of three experiments performed with similar results. qPCR analysis was performed to assess the expression of mRNA under normoxic and hypoxic conditions. For qPCR the data were normalized against GAPDH transcript as a reference gene and levels of RNA expression were determined with the 2^−ΔΔ*C*t^ method (* *p* < 0.05 vs. control).

## Abbreviations

CA	Caffeic Acid
Met	Metfomin
FA	Fatty Acids
AMPK	5′-adenosine monophosphate-activated protein kinase
ACC1	Acetyl-CoA Carboxylase 1
ACLY	ATP Citrate Lyase
FAS	Fatty Acid Synthase
SCD1	Stearoyl-CoA Desaturase-1
GLUT1	Glucose transporter 1
PDH	Pyruvate Dehydrogenase Complex
PDK	Pyruvate Dehydrogenase Kinase
GLS	Glutaminase
ME1	Malic Enzyme 1
ETC	Electron Transport Chain
OXPHOS	Oxidative Phosphorylation
ROS	Reactive Oxygen Species
PPP	Pentose Phosphate Pathway
NADPH	Nicotinamide adenine dinucleotide phosphate, reduced form
NADP	Nicotinamide adenine dinucleotide phosphate, oxidized form
NADPH	Nicotinamide adenine dinucleotide phosphate, reduced form
ATP	Adenosine triphosphate
AMP	Adenosine monophosphate
EMT	Epithelial-Mesenchymal Transition
VDAC	voltage-dependent anion channel
FAS receptor	first apoptosis signal receptor

## Appendix A

CA at concentrations ranged from 100 µM to 10 mM significantly inhibited proliferation of C-4I cervical carcinoma cells (*p* < 0.05 vs. untreated control for 100 µM, *p* < 0.01 vs. untreated control for 1 mM, 5 mM, 10 mM), as measured with MTT assay, and Met significantly decreased the viability of cells at 50 mM and 100 mM (*p* < 0.01 vs. untreated cells, Figure A1A,B, respectively). In present experiments CA at 100 µM and co-treatment of CA with Met at 10 mM inhibited proliferation (*p* < 0.05 vs. control and *p* < 0.01 vs. control) and growth of C-4I cells, compared with untreated control (Figure A1C,D, respectively). Likewise, the presence of CA and also the combination of CA and Met in culture medium caused cytotoxicity (*p* < 0.01 vs. control and *p* < 0.01 vs. control, Figure A1E) and aggravated cell morphology comparing to untreated cells, as shown in Figure A1F.
