# Supplementary material for: Caffeic Acid Targets AMPK Signaling and Regulates Tricarboxylic Acid Cycle Anaplerosis while Metformin Downregulates HIF-1α-Induced Glycolytic Enzymes in Human Cervical Squamous Cell Carcinoma Lines
Source: Nutrients. 2018 Jun 28;10(7):841. doi: 10.3390/nu10070841 (PMC6073805; doi:10.3390/nu10070841)
Supplement: Supplementary file 1 [file nutrients-10-00841-s001.zip › Supplement 1.docx]

**Supplement 1**

Figure S1. Met downregulates transcripts for glycolytic enzymes in HTB-35 cells. The cells were treated for 24 h with CA (100 μM) and/or Met (10 mM) either in normoxia (21% O_2_ level) or in hypoxia (5% O_2_ level). RT-PCR analysis was used and *HPRT1* was a reference gene. Experiments were repeated three times with similar results and presented as mean values ± SD.

RT-PCR revealed that Met suppressed the mRNA level of glycolytic regulatory proteins in HTB-35 cells during normoxia and hypoxia. In particular, Met decreased transcript for Glucose transporter 1 (*GLUT1*), Glucose transporter 3 (*GLUT3*), Hexokinase 2 (*HK2*), 6-Phosphofructo-2-Kinase/ Fructose-2,6-Biphosphatase 4 (*PFKFB4*), Pyruvate Kinase (*PKM),* Lactate Dehydrogenase A (*LDH*) and Pyruvate dehydrogenase kinase 1 (*PDK 1*) under hypoxic conditions. CA exerted weaker effect on expression of glycolytic regulatory genes in HTB-35 cell. We detected minor changes in the mRNA level for glycolytic enzymes in C4-I cells under CA, Met and co-treatment.
