# Supplementary material for: Caffeic Acid Targets AMPK Signaling and Regulates Tricarboxylic Acid Cycle Anaplerosis while Metformin Downregulates HIF-1α-Induced Glycolytic Enzymes in Human Cervical Squamous Cell Carcinoma Lines
Source: Nutrients. 2018 Jun 28;10(7):841. doi: 10.3390/nu10070841 (PMC6073805; doi:10.3390/nu10070841)
Supplement: Supplementary file 1 [file nutrients-10-00841-s001.zip › Supplement 2.docx]

**Supplement 2**

**Figure S2**. The effect of Metformin (Met) and Caffeic Acid (CA) on expression of *BAX,* *BCL-2, c-Myc* and *CCND1* genes in C-4I cells. The cells were incubated either in normoxia (21% O2 level) or in hypoxia (5% O2 level) with CA (100 μM), Met (10 mM) or both compounds for 24 h. CA inhibited the expression of *CCND1* and *c-Myc* and CA/Met suppressed *BCL-2* gene in HTB-35 cells (B). Data shown here are representative of three experiments performed with similar results. qPCR analysis was performed to assess the expression of mRNA under normoxic and hypoxic conditions. For qPCR the data were normalized against *GAPDH* transcript as a reference gene and levels of RNA expression were determined with the 2^−ΔΔ^*^C^*^t^ method (* *p* < 0.05 vs. control).

We used real-time PCR to analyze the effects of Caffeic Acid (CA) and Metformin (Met) at the mRNA level in C-4I cells. Under hypoxic and normoxic conditions CA increased the expression of pro-apoptotic *BAX* gene (Figure S1, *p<*0.05 vs. untreated control). CA/Met downregulated anti-apoptotic *BCL-2* gene in normoxia (*p<*0.05 vs. untreated control). CA, CA/Met suppressed the expression of cyclin-D1 (*CCND1)* (*p<*0.05 vs. untreated control) under normoxia (*p<*0.05 vs. untreated control). CA reduced the mRNA for *c-Myc* (*p*<0.05 vs. control) in C-4I cells exposed to hypoxia and normoxia as well (Figure S1, *p<*0.05 vs. untreated control).
