# Supplementary figures and images for: Caffeic Acid Targets AMPK Signaling and Regulates Tricarboxylic Acid Cycle Anaplerosis while Metformin Downregulates HIF-1α-Induced Glycolytic Enzymes in Human Cervical Squamous Cell Carcinoma Lines
Source: Nutrients. 2018 Jun 28;10(7):841. doi: 10.3390/nu10070841 (PMC6073805; doi:10.3390/nu10070841)

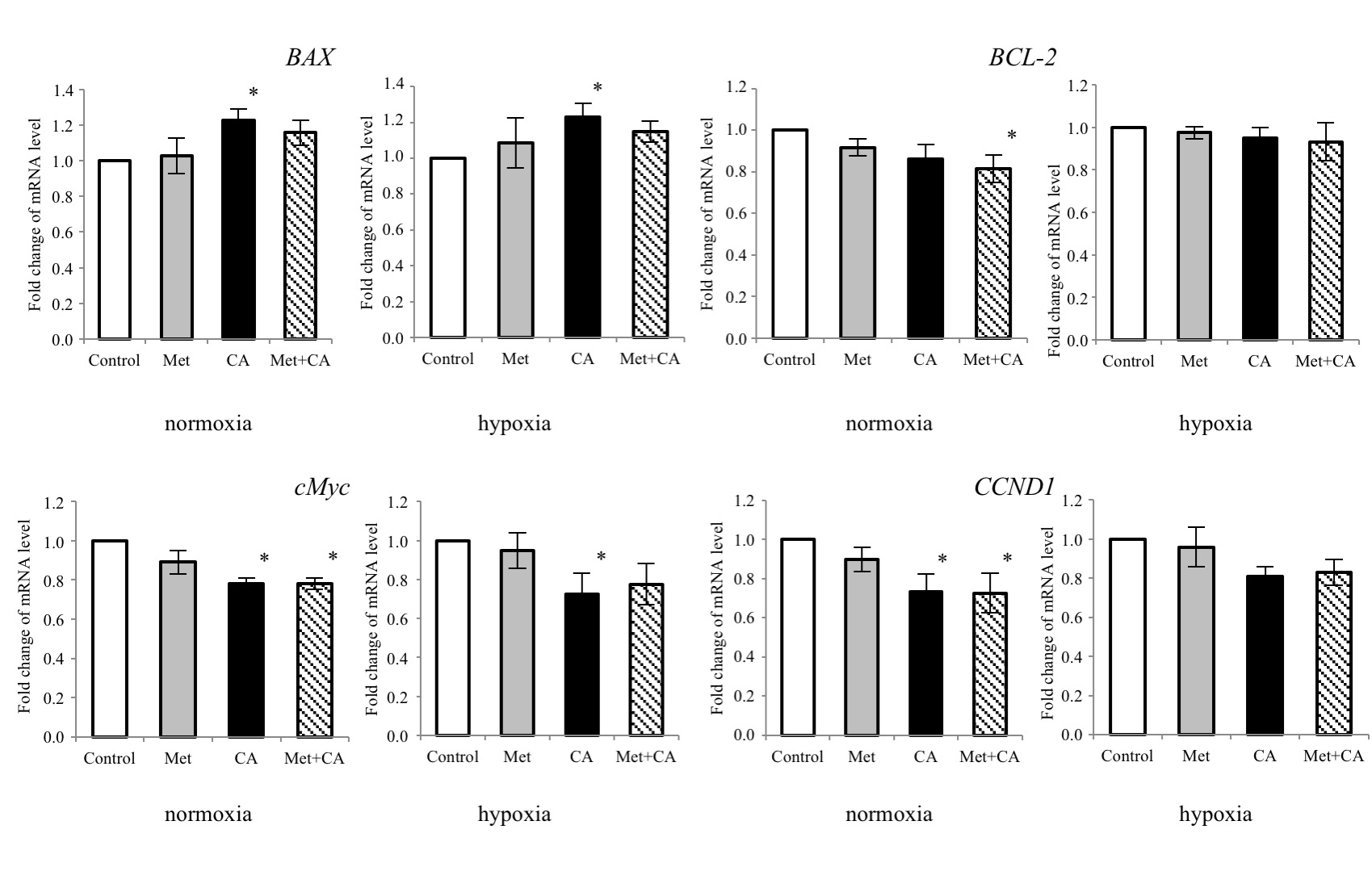

Supplement: Supplementary file 1 [file nutrients-10-00841-s001.zip › Fig S2.jpg]

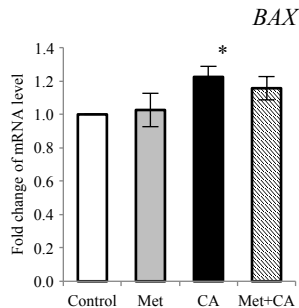

normoxia

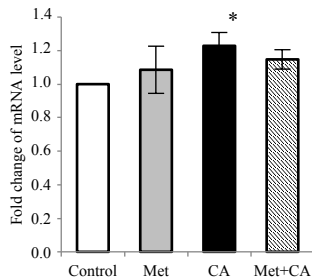

hypoxia

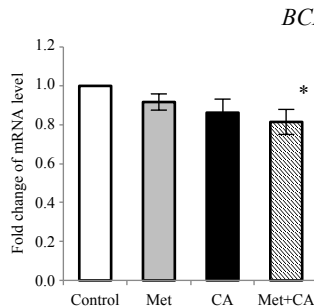

normoxia

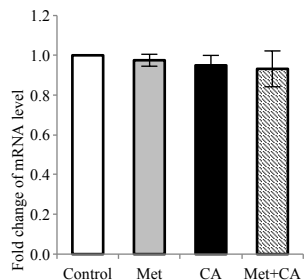

hypoxia

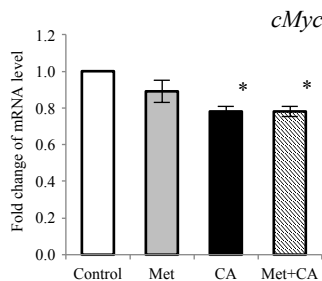

normoxia

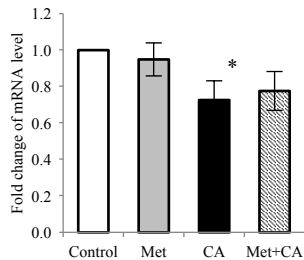

hypoxia

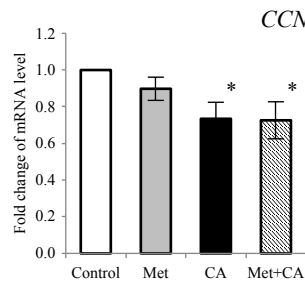

normoxia

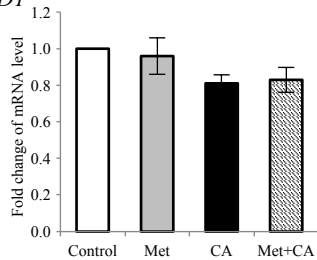

hypoxia

Supplement: Supplementary file 1 [file nutrients-10-00841-s001.zip › Fig S2.pdf]

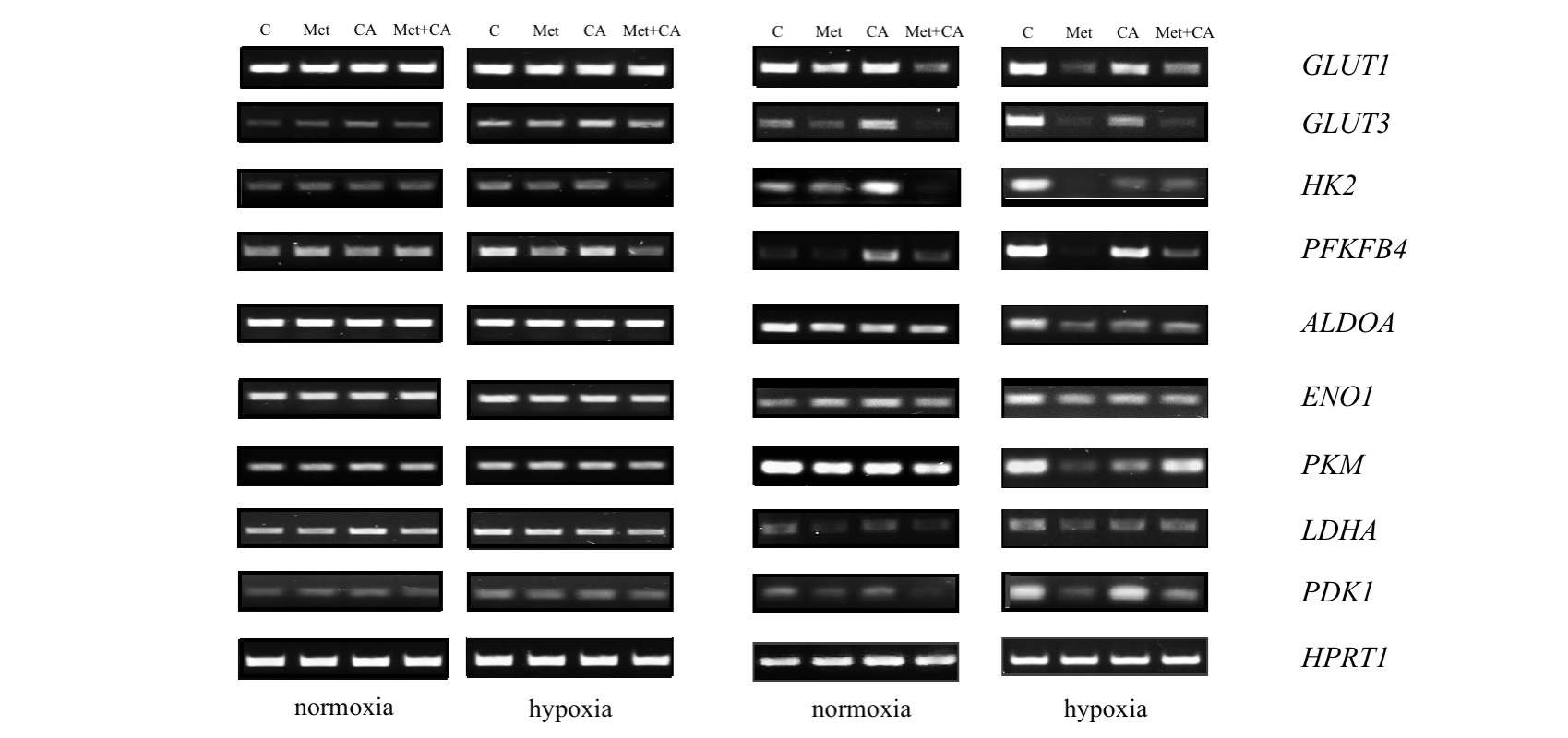

Supplement: Supplementary file 1 [file nutrients-10-00841-s001.zip › Fig S1.jpg]

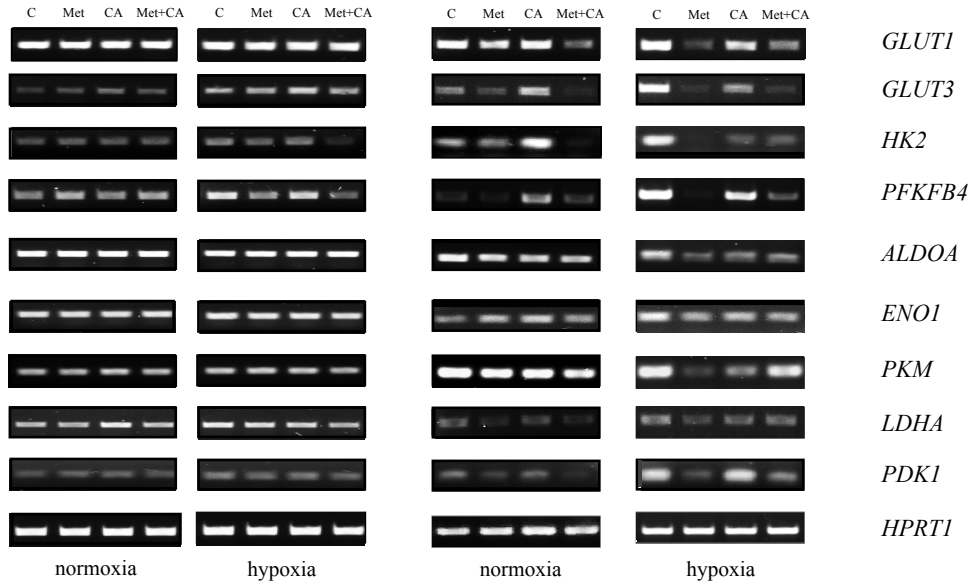

*GLUT1*

*GLUT3*

*HK2*

*PFKFB4*

ALDOA

*ENO1*

*PKM*

*LDHA*

*PDK1*

*HPRT1*

Supplement: Supplementary file 1 [file nutrients-10-00841-s001.zip › Fig S1.pdf]
